# Prevalence of asthma and allergies in children from the Greek-Cypriot and Turkish-Cypriot communities in Cyprus: a bi-communal cross-sectional study

**DOI:** 10.1186/1471-2458-13-585

**Published:** 2013-06-16

**Authors:** Demetris Lamnisos, Maria Moustaki, Ourania Kolokotroni, Huseyin Koksoy, Muharrem Faiz, Kenan Arifoglu, Donald K Milton, Nicos Middleton, Panayiotis K Yiallouros

**Affiliations:** 1 Cyprus International Institute for Environmental & Public Health in Association with Harvard School of Public Health, Cyprus University of Technology, Limassol, Cyprus; 2 Department of Nursing, School of Health Sciences, Cyprus University of Technology, Limassol, Cyprus; 3 3rd Department of Pediatrics, Attikon University Hospital, Athens, Greece; 4 Cyprus Turkish Medical Association, Nicosia, Cyprus; 5 Cyprus Social and Economic Research Centre – KADEM, Nicosia, Cyprus; 6 Harvard School of Public Health, Boston, Massachusetts, USA; 7 University of Maryland School of Public Health, College Park, Maryland, USA

**Keywords:** Asthma, Allergic rhinoconjuctivits, Eczema, Children, Cyprus, Greek Cypriot, Turkish Cypriot

## Abstract

**Background:**

The Greek-Cypriot (G/C) and Turkish-Cypriot (T/C) communities have lived apart since 1974, with the former presumably adopting a more westernized way of life. We estimated the prevalence of asthma and allergies among children in the two communities and investigated differences in socio-demographic and lifestyle risk factors.

**Methods:**

The ISAAC questionnaire was completed by 10156 children aged 7–8 and 13–14 years. Relative differences in asthma and allergic symptoms between the two communities were expressed as odds ratios (OR), estimated in multivariable logistic regression models before and after adjusting for participants’ risk characteristics.

**Results:**

In contrast to our original speculation, consistently lower prevalence rates were observed for respiratory outcomes (but not eczema) among G/C compared to T/C children in both age-groups. For instance, the prevalence of current wheeze among 7–8 year-olds was 8.7% vs 11.4% (OR = 0.74, 95%, CI: 0.61, 0.90) and of current rhinoconjuctivitis 2.6% vs 4.9% (OR = 0.52, 95% CI: 0.37, 0.71). Surprisingly, the proportion reporting family history of allergy was almost double in the G/C community. With the exception of early life nursery attendance, several protective factors were more prevalent amongst T/C, such as bedroom sharing, less urbanized environment and exposure to farm animals. In contrast, exposure to tobacco smoke was more frequent in the T/C community. Controlling for risk factors did not account for the observed lower prevalence of current wheeze (in the younger age-group) and rhinoconjuctivitis (in both age-groups) among G/C children while differences in the prevalence of eczema between the two communities were no longer statistically significant.

**Conclusions:**

A mixed picture of potential risk factors was observed in the two communities of Cyprus, not consistently favoring one over the other community since, for example, bedroom sharing and rural living but also exposure to tobacco smoke were more common among T/C children. Investigated risk factors do not fully account for the lower prevalence of asthma and allergies among G/C children, especially against a background of higher family history of allergy in this community.

## Background

Fifteen years ago, the ISAAC phase I study revealed a striking 20-fold international difference in the prevalence of asthma, eczema and rhinoconjuctivitis symptoms [1]. When phase III of the ISAAC study re-assessed five years later the prevalence of the same conditions, the magnitude of observed differences, even though reduced, still remained wide [2,3]. It is now widely accepted that environmental exposures associated with westernized lifestyle are critically implicated in the development of allergic symptoms [4,5]. The fact that immigrants to more affluent countries initially have an innate advantage for allergic diseases, which nevertheless attenuates in the next generations can be interpreted in this context [6-8]. Furthermore, genetically similar populations exposed to different environmental conditions display different temporal trends in the prevalence of allergic symptoms. The most characteristic example is that of West and East Germany before unification. Compared to the 150% increase recorded in West Germany over the pre-unification period, the increase in East Germany during the same period was only marginal. The difference gradually narrowed after re-unification [9-11].

An interesting paradigm is encountered on the island of Cyprus, where the Greek-Cypriot (G/C) and Turkish-Cypriot (T/C) communities have been living apart since 1974, with the former having a higher average income per capita and presumably adopting a more “westernized” way of life. In the G/C community, childhood allergic diseases were recently reported to be rising [12]. Data from the T/C community are limited to a single epidemiological study published more than 10 years ago [13]. Despite sharing a small island of 9250 km^2^, the extent to which potential differences in socio-demographic and lifestyle factors between the two communities may have contributed to a different pattern in terms of childhood allergic conditions epidemiology is largely unknown.

The aim of this study was to provide the first bi-communal study using standardized methodology in order to (a) investigate the prevalence of asthma and allergy symptoms on both sides of the division line in Cyprus and (b) explore the extent to which differences in socio-demographic and other risk characteristics explain any observed differences between the two communities.

## Methods

### Study population and design

Children aged 7–8 and 13–14 years in both communities were contacted at the school setting during 2007–2009. In order to retain comparability with previous studies in the G/C community, schools in two out of the four districts controlled by the Republic of Cyprus (i.e. Nicosia and Limassol) were invited to participate (n = 124, 98% of those contacted) [12]. In the T/C community, schools from the whole geographical area north of the division line were invited to participate (n = 105, 100% of those contacted). Trained field workers in both communities distributed the respective approved Greek and Turkish version of the International Study of Asthma and Allergy in Childhood (ISAAC) core questionnaire, supplemented with questions on demographic and lifestyle characteristics. The questionnaire was self-completed by the older children (as per the ISAAC protocol) and by the parents of children in the case of the younger age-group. The study was approved by the Cyprus Bioethics Committee (EEBK/EP/2006/28). Informed written parental consent was obtained in all cases.

### Definition of outcome variables

Based on answers given to questions of the ISAAC questionnaire, we studied the following outcomes as defined previously [1,2]: *Current wheeze, Severity of asthma, Current eczema, and Current allergic rhinoconjuctivitis.* We have also explored potential differences in diagnosis of asthma, hay fever and eczema as well as report of ever wheeze between children from the two communities, but we chose to focus the analysis to reported symptoms rather than diagnostic labels (i.e. report of diagnosis of asthma, hay fever or eczema) in order to avoid confounding effects relating to possible differential use and/or understanding of diagnostic terms between the two communities.

While the Greek version of the ISAAC questionnaire has not been officially validated, the translation used in this study is the only available Greek version of the ISAAC core questionnaire, which was adopted by the ISAAC study and has been used by the two ISAAC centers in Greece (Athens, Thessaloniki). Furthermore, it has been used ever since as a standard tool in several studies in Greece, including the PANACEA epidemiological study [14], and it has been recently shown that reported symptoms correlate well with objective measures of allergy (skin prick testing) [15]. The Turkish version of the ISAAC questionnaire was tested in a pilot study conducted three months before the ISAAC study. The parental self-administered questionnaire and the face-to-face interview with the parents were found to be concordant [16].

### Definition of predictor variables

In order to describe the socio-demographic characteristics and the prevalence of risk factors of the sample participants from the two communities, the following variables were selected a priori: gender, nationality (defined as: “Both parents are Cypriots (either G/C or T/C)” vs “At least one parent is not Cypriot”), family history of allergies (i.e. positive report of asthma, eczema or hay fever in any sibling and/or parent), passive smoking (categorized as: none, up to 20, and more than 20 cigarettes smoked daily in the house), parental level of education (categorized in terms of the highest level of education attained by at least one of the parents), ownership of pets or other animals kept in the household or yard of the house, exposure to any farm animal (i.e. sheep and/or cow) at the house environment, number of older siblings and area of residence. Area of residence was characterized as urban if the family address was located within the boundaries or in the wider metropolitan areas in geographic continuity with one of the main cities on the island. If the family address was in a settlement not in geographic continuity with any of the cities, it was classified as semi-rural or rural depending on whether the population exceeded or not 1500 residents respectively. Parents of the 7–8 year-old participants also provided information on maternal smoking habits during pregnancy of the participating child, mode of delivery, birth weight, duration of exclusive breastfeeding, nursery attendance in first year of life, bedroom sharing up to age of 5 years while children in the older age group were only asked about their own smoking habits.

### Statistical analyses

For each community and age-group, prevalence estimates and 95% confidence intervals (CI) of asthma and allergic symptoms were estimated, as per ISAAC protocol [1,2]. In order to investigate differences between the two communities, the relative differences in the prevalence outcomes were expressed as odds ratios (OR) with their corresponding 95% CI, as estimated in logistic regression models. Multivariable models were used to investigate the extent to which differences in socio-demographic and other risk factors between the participating children from the two communities account for any observed differences in the study outcomes. A hierarchical approach was used whereby the crude estimates (model 1) were adjusted for socio-demographic characteristics and a priori selected main risk factors for asthma/allergy, excluding (model 2) and including (model 3) family history of allergy. Post-hoc, this proved particularly important due to the striking difference observed in self-reported family history between the two communities. In order to explore whether the strength of the association between any of the investigated factors (including family history of allergy) and study outcomes was differential in the two communities, models include an interaction term between community and each factor in turn. Statistical significance of the interaction terms was assessed in Likelihood Ratio Tests (LRT) for effect modification comparing models with and without the interaction terms. All analyses were performed using the statistical software R (version 2.12.1).

## Results

### Population characteristics

In total, 4569 children aged 7–8 years (2216 G/C and 2353 T/C, 48% and 65% response rate respectively) and 5587 children aged 13–14 years (2452 G/C and 3135 T/C, 44% and 87% response rate respectively) participated in the study. Table 1 presents the socio-demographic and other risk characteristics of the G/C and T/C children, revealing a different profile in terms of almost all the parameters examined.

**Table 1:** Socio-demographic and other risk characteristics of the participating children aged 7–8 and 13–14 from the Greek-Cypriot and Turkish-Cypriot communities

	**Children aged 7-8**			**Children aged 13-14**		
**Participant characteristics**	**G/C**	**T/C**	**p**^**†**^	**G/C**	**T/C**	**p**^**†**^
**(expressed as percentages)**	**(n = 2216)**	**(n = 2353)**		**(n = 2452)**	**(n = 3135)**	
Female gender (%)	49.5	50.6		48.2	51.7	
Parental education (%)						
Only one secondary education	5.3	49.9		13.0	43.0	
Both secondary education	40.9	30.3		46.7	36.8	
Only one tertiary education	24.1	12.8		21.2	11.6	
Both tertiary education	29.8	7.1	*	19.0	8.6	*
Area of residence (%)						
Urban	66.5	40.9		65.9	46.5	
Sub-rural	21.0	24.3		18.2	22.1	
Rural	12.5	34.8	*	15.9	31.4	*
Family history of allergies (%)	37.0	19.5	*	16.5	10.9	*
Older siblings (%)						
None	38.7	50.8		26.3	44.4	
1	34.3	34.3		37.8	34.4	
≥2	27.0	14.9	*	35.9	21.2	*
Passive smoking (%)						
Not at all	60.1	46.7		49.7	43.1	
Up to 20 cigarettes/day	25.3	40.3		35.5	48.3	
More than 20 cigarettes/day	14.7	13.0	*	14.8	8.6	
Animals at home/yard (%)	42.6	32.4	*	56.2	48.0	*
Farm animal at home/yard (%)	0.6	4.7	*	0.5	4.2	*
Caesarian delivery (%)^‡^	31.7	39.0	*	- - -	- - -	
Birth weight <2500gr (%)^‡^	13.9	18.0	*	- - -	- - -	
Duration of breastfeeding (%)^‡^						
Not at all	46.8	21.8		- - -	- - -	
<4 months	32.5	24.2		- - -	- - -	
≥4 months	20.6	54.0	*	- - -	- - -	
Bedroom sharing (%)^‡^	37.4	52.3	*	- - -	- - -	
Nursery in first year of life (%)^‡^	9.3	4.7	*	- - -	- - -	
Maternal smoking during pregnancy (%)^‡^	2.9	10.8	*	- - -	- - -	
Active smoking (%)^‡^	- - -	- - -		1.7	1.3	

* P-value of χ^2^ test comparing the G/C and T/C communities <0.01.

‡ Information on these variables was not obtained from the 13–14 year-old group, while active smoking was not included in the 7–8 year-old questionnaires.

As many as 59.1% of 7–8 year old T/C resided in less urbanized environments, 52.3% reported bedroom sharing in first 5 years of life and 4.7% reported keeping a farm animal in the home environment, as opposed to 33.5%, 37.4% and 0.6% respectively amongst G/C children (all p < 0.01). While many presumably protective factors as per the hygiene hypothesis were more prevalent in the T/C community, nursery attendance in first year of life (9.3% vs 4.7%, p < 0.01) and presence of older siblings (62.3% vs 49.2%, p < 0.01) were higher among 7–8 year-old G/C children. Furthermore, more T/C children were exclusively breastfed (78.2% vs 53.7%, p < 0.01) while rates of caesarian delivery were similarly high in both communities (39% vs 31.7%, p < 0.01), even though the difference was statistically significant due to the large size of the survey.

In contrast, exposure to tobacco smoke was higher in the T/C community, both as passive smoking at the time of survey (53.3% vs 39.9%, p < 0.01) as well as during pregnancy (10.8% vs 2.9%, p < 0.01). A similar picture emerged in the 13–14 year old group. Finally, as expected, a higher level of educational attainment was recorded amongst parents in the G/C community. In contrast, the proportion reporting family history of allergies was strikingly higher in the G/C community with 37% vs 19.5%, (p < 0.01) in the younger and 16.5% vs 10.9% (p < 0.01) in the older age-group.

### Prevalence of asthma and allergic symptoms in the G/C and T/C communities

**Figure 1:**
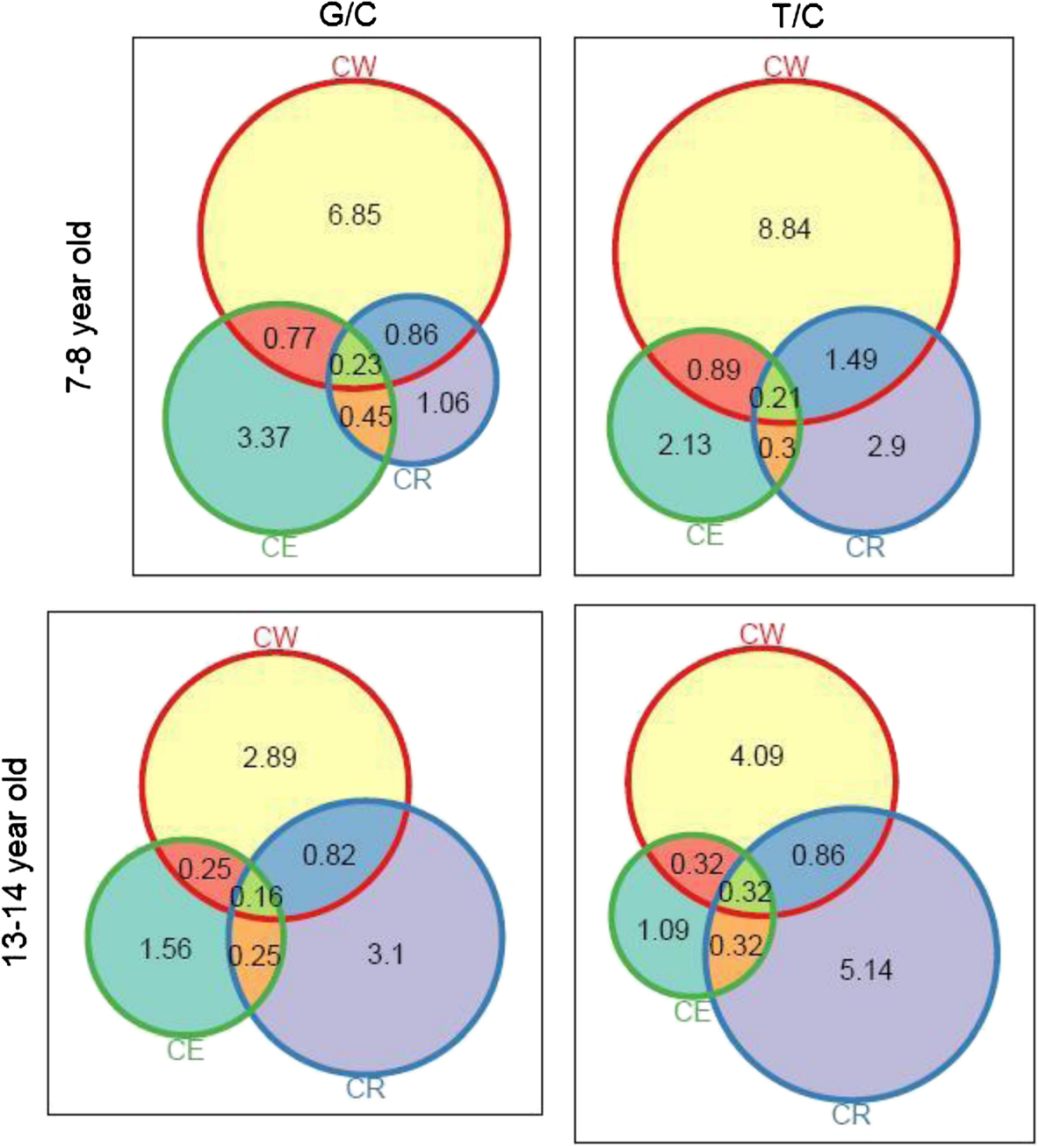
**Proportional Venn diagrams of the prevalence of asthma and allergic symptoms in children of the G/C and T/C communities.** Proportional Venn diagrams presenting the prevalence of current wheeze (CW), current allergic rhinoconjuctivitis (CR) and current eczema (CE) in 7–8 and 13–14 year-old children from the Greek-Cypriot and Turkish-Cypriot communities.

Table 2 presents age- and community-specific prevalence estimates of asthma and allergy symptoms. To better visualize the prevalence estimates in the two communities for individual study outcomes, as well as their interrelationships, proportional Venn diagrams are presented in Figure 1. Current eczema was far more prevalent among young G/C children compared to T/C children, while the reverse is true for rhinoconjuctivitis. In the older age-group, while the prevalence of eczema appears similar in the two communities, the prevalence of rhinoconjuctivitis remains more prevalent among T/C children.

**Table 2:** The prevalence of asthma and allergic symptoms among children aged 7–8 and 13–14 from the Greek-Cypriot and Turkish-Cypriot communities along with unadjusted and adjusted odds ratios comparing the two communities

**Outcome**	**G/C**	**T/C**	***G/C Vs T/C***		
	**Prevalence**	**Prevalence**	**Unadjusted**	**Adjusted OR†**	***Adjusted OR‡***
	**(95% CI)**	**(95% CI)**	**OR (95% CI)**	**(95% CI)**	***(95% CI)***
***Children 7–8 years of age***					
Current Wheeze	8.71	11.43	0.74	0.73	*0.84*
	(7.60, 9.96)	(10.21, 12.78)	(0.61, 0.90)	(0.54, 0.98)	*(0.63, 1.12)*
Severe Asthma	2.61	4.93	0.52	0.67	*0.78*
	(2.02, 3.37)	(4.13, 5.88)	(0.37, 0.71)	(0.41, 1.07)	*(0.49, 1.24)*
Current Rhinoconjuctivitis	2.60	4.90	0.52	0.61	*0.70*
	(2.00, 3.36)	(4.09, 5.84)	(0.37, 0.71)	(0.38, 0.99)	*(0.43, 1.12)*
Current Eczema	4.72	3.53	1.36	1.22	*1.38*
	(3.91, 5.69)	(2.85, 4.35)	(1.01, 1.83)	(0.79, 1.91)	*(0.90, 2.13)*
***Children 13–14 years of age***					
Current Wheeze	4.12	5.59	0.73	0.76	*0.83*
	(3.40, 4.99)	(4.84, 6.46)	(0.56, 0.93)	(0.54, 1.05)	*(0.60, 1.14)*
Severe Asthma	1.82	2.97	0.61	0.52	*0.62*
	(1.36, 2.43)	(2.43, 3.62)	(0.42, 0.87)	(0.34, 0.85)	*(0.38, 0.99)*
Current Rhinoconjuctivitis	4.33	6.64	0.64	0.72	*0.76*
	(3.59, 5.22)	(5.81, 7.58)	(0.50, 0.81)	(0.52, 1.00)	*(0.55, 1.06)*
Current Eczema	2.22	2.05	1.09	1.21	*1.29*
	(1.71, 2.89)	(1.61, 2.61)	(0.75, 1.56)	(0.73, 2.00)	*(0.78, 2.14)*

*† For 7–8 years old:* Adjusted for sex, parent’s level of education, area of residence, family history of allergy, number of siblings, passive smoking, animals at home, maternal smoking during pregnancy, mode of delivery, birth weight, exclusive breastfeeding duration and bedroom sharing. *For 13–14 years old:* Adjusted for sex, parent’s level of education, area of residence, family history of allergy, number of siblings, passive and active smoking and animals at home.

*‡* Adjusted for all the above participant characteristics *except family history of allergy.*

With the exception of eczema, consistently lower prevalence for all respiratory outcomes was observed among children in the G/C community in both age-groups. The prevalence of current wheeze among 7–8 year-olds was 8.7% in G/C vs 11.4% in T/C (OR: 0.74, 95% CI: 0.61-0.90) whereas the equivalent figures among 13–14 year-olds were 4.1% vs 5.6% (OR 0.73, CI: 0.56-0.95). Similarly, the prevalence of severe asthma among younger children was 2.6% vs 4.9% (OR 0.52, CI: 0.37-0.71) and 1.8% vs 3.0% (OR 0.61, CI: 0.42-0.87) in the older children. After controlling for participants’ characteristics in multivariable models, there was only slight attenuation in the estimates. However, while adjusted Odds Ratios (aORs) were lower than 1 for all outcomes, only current wheeze in the younger age-group (aOR 0.73, CI: 0.54-0.98) and severe asthma in the older age-group (aOR 0.52, CI: 0.34-0.85) remained statistically significant. Adjusted ORs for severe asthma in the younger age-group and current wheeze in the older age-group were no longer statistically significant at the 5% level.

Similarly, a lower prevalence of current rhinoconjuctivitis was also observed in G/C community, 2.6% vs 4.9% among the 7–8 year olds and 4.3% vs 6.6% among 13–14 year olds, while in this case the community effect remained significant even after controlling for the different risk characteristics of the samples (aOR 0.61, CI: 0.38-0.99 and aOR 0.72, CI: 0.52-1.00 respectively).

In contrast, a slightly higher prevalence of current eczema symptoms was found among the 7–8 year-olds in G/C community, 4.7% vs 3.5% (OR 1.36, CI: 1.01-1.83), while no difference was observed in the older age-group. Nevertheless, the aOR for current eczema were not statistically significant in either group suggesting no independent community effect on the prevalence of eczema after controlling for risk factors.

In a subgroup analysis of children whose parents are both of Cypriot origin (i.e. either both G/C or both T/C in each community respectively) the results remained largely unchanged (see Table 3). The prevalence estimates in the T/C community decreased for all study outcomes, and as a result the gap between the two communities appeared smaller (or bigger in the case of eczema). While this is suggestive of higher prevalence among the children in the T/C community whose parents are not both of T/C origin (e.g. immigrant groups), the prevalence estimates in the T/C are still higher for all study outcomes (other than eczema) whereas confidence intervals become wider as a result of the reduced sample size.

**Table 3:** The prevalence of asthma and allergic symptoms among children aged 7–8 and 13–14 from the Greek-Cypriot and Turkish-Cypriot communities, restricting the analysis to those children whose parents are both of Cypriot origin, along with unadjusted and adjusted odds ratios comparing the two communities

**Outcome**	**G/C**	**T/C**	***G/C Vs T/C***		
	**Prevalence**	**Prevalence**	**Unadjusted**	**Adjusted OR†**	***Adjusted OR‡***
	**(95% CI)**	**(95% CI)**	**OR (95% CI)**	**(95% CI)**	***(95% CI)***
***Children 7–8 years of age***					
Current Wheeze	8.94	10.91	0.80	0.78	0.93
	(7.70, 10.36)	(8.95, 13.23)	(0.61, 1.06)	(0.54, 1.14)	(0.65, 1.33)
Severe Wheeze	2.56	3.55	0.71	0.82	0.96
	(1.92, 3.41)	(2.49, 5.06)	(0.45, 1.16)	(0.44, 1.55)	(0.53, 1.79)
Current Rhinoconjuctivitis	2.60	3.20	0.81	0.88	1.01
	(1.95, 3.46)	(2.19, 4.64)	(0.50, 1.33)	(0.46, 1.71)	(0.53, 1.93)
Current Eczema	5.00	3.31	1.54	1.41	1.60
	(4.08, 6.12)	(2.28, 4.77)	(1.00, 2.43)	(0.81, 2.53)	(0.93, 2.84)
***Children 13–14 years of age***					
Current Wheeze	3.99	4.85	0.82	0.97	1.02
	(3.23, 4.91)	(3.80, 6.17)	(0.58, 1.14)	(0.65, 1.44)	(0.69, 1.51)
Severe Wheeze	1.85	2.43	0.76	0.76	0.84
	(1.36, 2.53)	(1.72, 3.43)	(0.47, 1.23)	(0.42, 1.38)	(0.47, 1.49)
Current Rhinoconjuctivitis	4.40	3.90	1.14	1.07	1.08
	(3.60, 5.37)	(2.96, 5.11)	(0.80, 1.63)	(0.71, 1.64)	(0.71, 1.64)
Current Eczema	2.17	1.00	2.18	1.79	1.86
	(1.63, 2.89)	(0.59, 1.72)	(1.21, 4.22)	(0.91, 3.70)	(0.96, 3.83)

*† For 7–8 years old:* Adjusted for sex, parent’s level of education, area of residence, family history of allergy, number of siblings, passive smoking, animals at home, maternal smoking during pregnancy, mode of delivery, birth weight, exclusive breastfeeding duration and bedroom sharing. *For 13–14 years old: Adjusted* for sex, parent’s level of education, area of residence, family history of allergy, number of siblings, passive and active smoking and animals at home.

*‡* Adjusted for all the above participant characteristics *except family history of allergy.*

**Figure 2:**
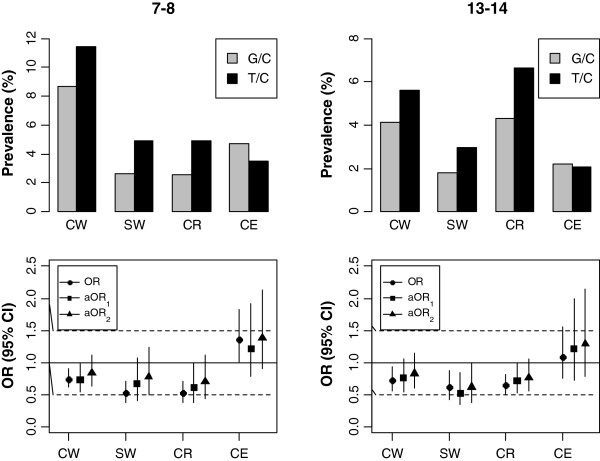
**Prevalence and adjusted odds ratios of asthma and allergic symptoms in children of the G/C and T/C communities.** Prevalence and odds ratios of asthma and allergic symptoms in 7–8 and 13–14 year old children from the Greek-Cypriot as compared to the Turkish-Cypriot community before and after adjusting for potential risk factors (including and excluding family history of allergies).

The prevalence estimates, as well as the effect of adjusting for differences in the socio-demographic and other risk characteristics of the participants are also presented graphically in Figure 2. It is apparent that the G/C vs T/C odds ratios appear slightly stronger in the case of eczema and attenuate in the case of all other outcomes after removing family history of allergies from the multivariable models. The only exception was the case of severe asthma in the older age-group which still remains significantly less prevalent in the G/C community (aOR 0.62, CI: 0.38-0.99). This suggests that the community effect in G/C appears particularly favorable compared to the T/C for wheeze and rhinoconjuctivitis given the much higher prevalence of reported family predisposition amongst G/C children.

Despite the much lower reported prevalence of family history of allergy among parents of T/C children, a consistently higher prevalence of diagnosis of asthma (19.3% vs 17.8% in children aged 7–8 and 15.4% vs 11.3% in children aged 13–14) and hay-fever (11.6% vs 5.4% in children aged 7–8 and 10.2% vs 2.9% in children aged 13–14) was observed in the T/C community. The only exception was the diagnosis of eczema which was also the only symptom which exhibited higher prevalence in the G/C community.

### Association of asthma and allergic symptoms with predictors involved in the hygiene hypothesis

We observed associations between several factors projected in the hygiene hypothesis with asthma and allergic symptoms but these were not consistent across all study outcomes or both age-groups (see Table 4). For instance, adjusted ORs for current wheeze (aOR: 0.72, 95% CI: 0.53, 0.98), severe asthma (aOR: 0.56, 95% CI: 0.56, 0.91) and current eczema (aOR:0.36, 95% CI: 0.20, 0.61) among children aged 7–8 years with two or more older siblings suggested a protective effect. Nevertheless, this was not generally the case with the older age-group. While no overall effect was observed in this age-group, there was some evidence to suggest that the protective effect of sibling order, at least for wheezing, may be restricted to G/C children (LRT p-value for effect modification with community < 0.05). In contrast, nursery attendance in first year of life was not associated with any of the study outcomes while bedroom sharing was associated with an elevated risk for severe asthma among the younger age-group (aOR 1.69, 95% CI: 1.17, 2.43). Furthermore, exposure to farm animals was not associated with any of the study outcomes in any age-group while rural environment appeared protective for wheezing, but not eczema and rhinoconjuctivitis, only in the older age group. In the case of rhinoconjuctivitis, there was some evidence that a rural environment may be protective amongst older G/C children but, if anything, the opposite was true in the case of T/C children. Other than the few exceptions mentioned here, there was generally no statistical evidence that the strength of association between any of these risk factors and study outcomes differed by community.

**Table 4:** Unadjusted and adjusted odds ratios of asthma and allergic symptoms in children aged 7–8 and 13–14 from the Greek-Cypriot and Turkish-Cypriot communities in relation to risk factors projected in the hygiene hypothesis

	***Children 7–8 years of age***							
	**Current wheeze**		**Severe asthma**		**Current eczema**		***Current Rhinoconjuctivitis***	
	**Unadjusted OR (95%CI)**	**Adjusted OR† (95% CI)**	**Unadjusted OR (95% CI)**	**Adjusted OR† (95% CI)**	**Unadjusted OR (95% CI)**	**Adjusted OR† (95% CI)**	**Unadjusted OR(95% CI)**	***Adjusted OR† (95% CI)***
**Older sibling**								
1 (Vs None)	0.86 (0.69, 1.07)	0.76 (0.59, 0.97)	1.08 (0.77, 1.51)	0.80 (0.55, 1.16)	0.87 (0.63, 1.20)	0.70 (0.48, 1.00)	0.87 (0.61. 1.23)	*0.76 (0.51, 1.11)*
≥2 (Vs None)	0.83 (0.63, 1.08)	0.72 (0.53, 0.98)	0.82 (0.52, 1.27)	0.56 (0.33, 0.91)	0.48 (0.29, 0.75)	0.36 (0.20, 0.61)	0.97 (0.63, 1.46)	*0.77 (0.47, 1.22)*
**Farm animal at home**	1.11 (0.62, 1.87)	0.99 (0.54, 1.72)	1.13 (0.46, 2.36)	0.97 (0.38, 2.10)	1.26 (0.48, 2.74)	1.33 (0.49, 3.04)	1.26 (0.52, 2.62)	*1.02 (0.40, 2.21)*
**Area of residence**								
Sub-rural (Vs Rural)	1.04 (0.79, 1.37)	1.09 (0.82, 1.45)	1.40 (0.92, 2.13)	1.49 (0.96, 2.31)	1.01 (0.64, 1.61)	1.12 (0.69, 1.80)	1.03 (0.67, 1.57)	*1.08 (0.70, 1.68)*
Urban (Vs Rural)	0.87 (0.69, 1.12)	0.87 (0.67, 1.13)	1.04 (0.70, 1.54)	1.02 (0.67, 1.56)	1.20 (0.82, 1.79)	1.20 (0.79, 1.85)	0.89 (0.61, 1.30)	*0.83 (0.56, 1.26)*
**Bedroom sharing**	1.09 (0.90, 1.33)	1.20 (0.95, 1.52)	1.61 (1.18, 2.20)	1.69 (1.17, 2.43)	1.00 (0.75, 1.36)	1.29 (0.90, 1.86)	1.16 (0.85, 1.58)	*1.17 (0.82, 1.69)*
**Nursery attendance**	1.25 (0.84, 1.96)	1.28 (0.85, 2.03)	0.81 (0.47, 1.53)	0.85 (0.49, 1.62)	0.88 (0.53, 1.58)	0.84 (0.49, 1.52)	0.97 (0.55, 1.92)	*1.05 (0.58, 2.10)*
	***Children 13–14 years of age***							
	**Current wheeze**		**Severe asthma**		**Current eczema**		***Current Rhinoconjuctivitis***	
	**Unadjusted OR (95%CI)**	**Adjusted OR***‡***(95% CI)**	**Unadjusted OR (95% CI)**	**Adjusted OR***‡***(95% CI)**	**Unadjusted OR (95% CI)**	**Adjusted OR***‡***(95% CI)**	**Unadjusted OR (95% CI)**	***Adjusted OR****‡****(95% CI)***
**Older sibling**								
1 (Vs None)	0.87 (0.65, 1.16)	0.87 (0.64, 1.19)	1.00 (0.67, 1.48)	1.03 (0.66, 1.58)	0.86 (0.54, 1.37)	0.84 (0.50, 1.39)	1.33 (1.00. 1.77)	*1.29 (0.95, 1.76)*
≥2 (Vs None)	0.82 (0.59, 1.13)	0.90 (0.64, 1.26)	0.86 (0.53, 1.35)	0.95 (0.57, 1.55)	1.43 (0.92, 2.24)	1.40 (0.86, 2.27)	1.59 (1.17, 2.15)	*1.41 (1.02, 1.96)*
**Farm animal at home**	0.35 (0.04, 0.94)	0.39 (0.10, 1.05)	0.94 (0.28, 2.30)	0.78 (0.19, 2.19)	0.88 (0.21, 2.42)	0.94 (0.23, 2.66)	1.15 (0.58, 2.08)	*1.03 (0.49, 1.94)*
**Area of residence**								
Sub-rural (Vs Rural)	1.52 (1.05, 2.20)	1.64 (1.11, 2.42)	1.61 (0.97, 2.71)	1.77 (1.02, 3.10)	0.99 (0.56, 1.72)	1.04 (0.56, 1.88)	0.68 (0.47, 0.96)	*0.75 (0.51, 1.10)*
Urban (Vs Rural)	1.37 (1.00, 1.90)	1.44 (1.02, 2.05)	1.42 (0.91, 2.26)	1.46 (0.90, 2.43)	1.03 (0.66, 1.65)	1.29 (0.80, 2.12)	0.89 (0.68, 1.16)	*0.98 (0.73, 1.33)*

† Adjusted for each other as well as community (G/C and T/C), gender, parents’ nationality, parents’ level of education, family history of allergies, animals at home, passive smoking, maternal smoking during pregnancy, mode of delivery, birth weight and exclusive breastfeeding duration.

‡ Adjusted for each other as well as community (G/C and T/C), gender, parents’ nationality, parents’ level of education, family history of allergies, animals at home and passive smoking at home. Bedroom sharing, nursery attendance, maternal smoking during pregnancy, mode of delivery, birth weight and exclusive breastfeeding duration were not available for the 13–14 year-old children.

### Association of asthma and allergic symptoms with other risk characteristics

Table 5 presents the unadjusted and adjusted ORs of asthma and allergic symptoms with other potential risk factors. As expected, allergic background was generally the strongest and most consistent predictor of reported symptoms, with aORs of a magnitude of 2 to 5 across all outcomes in both age-groups. In the older age-group, active smoking was also a strong predictor with aORs of 4.49 (95% CI: 2.17, 8.64) for current wheeze, 6.03 (95% CI: 2.46, 13.17) for severe asthma and 2.60 (95% CI: 1.15, 5.27) for current rhinoconjuctivitis. Having animal(s) at home was a significant risk factor for current eczema (aOR: 2.35, 95% CI: 1.54, 3.65) and current rhinoconjuctivitis (aOR:1.40, 95% CI:1.08, 1.82) only in the older children. Similarly, exposure to passive smoking in the household also carried a more pronounced effect in the older age-group, with significant associations with current wheeze (aOR:1.57, 95% CI: 1.04, 2.32), current eczema (aOR: 1.81, 95% CI: 1.02, 3.13) and current rhinoconjuctivitis (aOR: 2.73, 95% CI: 1.91, 3.89) in those exposed to 20 or more cigarettes’ smoke daily. Some statistical evidence of effect modification by community was observed for exposure to tobacco smoke in the younger age-group, where association with wheeze was only observed amongst T/C (aOR: 1.56, 95% CI: 1.19, 2.05) but not G/C children (aOR: 0.86, 95% CI: 0.59, 1.22). Exposure to maternal smoking during pregnancy was also found to be associated with current wheeze (aOR: 1.55, 95% CI: 1.08, 2.17) and current eczema (aOR: 1.84, 95% CI: 1.04, 3.11) in the younger children. In the case of eczema, the association with maternal smoking during pregnancy is restricted to T/C children (aOR: 2.44, 95% CI: 1.31, 4.35) since a similar effect was not observed among G/C children (aOR: 0.31, 95% CI: 0.02, 1.57). Finally, the level of parental education did not appear to be associated with any of the study outcomes among the older children, whereas in the younger children associations were sporadic and no suggestive of a clear pattern. Other than the few exceptions mentioned here, there was generally no statistical evidence that different factors were important in the two communities or that the strength of association between any of these risk factors and the study outcomes differed by community.

**Table 5:** Unadjusted and adjusted odds ratios of asthma and allergic symptoms in children aged 7–8 and 13–14 from the Greek-Cypriot and Turkish-Cypriot communities in relation to other risk factors in G/C and T/C children

	***Children 7–8 years of age***							
	**Current wheeze**		**Severe asthma**		**Current eczema**		***Current Rhinoconjuctivitis***	
	**Unadjusted OR (95%CI)**	**Adjusted OR† (95% CI)**	**Unadjusted OR (95% CI)**	**Adjusted OR† (95% CI)**	**Unadjusted OR (95% CI)**	**Adjusted OR† (95% CI)**	**Unadjusted OR (95% CI)**	***Adjusted OR† (95% CI)***
**Parental education**								
Both secondary	0.77 (0.59, 1.00)	0.77 (0.57, 1.02)	0.60 (0.40, 0.89)	0.67 (0.43, 1.03)	1.03 (0.67, 1.59)	0.92 (0.58, 1.47)	0.44 (0.28, 0.67)	*0.50 (0.31, 0.79)*
At most one tertiary	0.70 (0.50, 0.96)	0.63 (0.44, 0.90)	0.48 (0.27, 0.80)	0.45 (0.25, 0.78)	1.03 (0.62, 1.70)	0.80 (0.46, 1.37)	0.71 (0.43, 1.13)	*0.74 (0.44, 1.22)*
Both tertiary	1.27 (0.93, 1.73)	1.13 (0.79, 1.61)	0.84 (0.50, 1.37)	0.80 (0.45, 1.39)	1.55 (0.95, 2.53)	1.01 (0.58, 1.76)	0.87 (0.53, 1.42)	*0.86 (0.49, 1.51)*
**Animals at home**	1.15 (0.94, 1.41)	1.13 (0.91, 1.40)	1.11 (0.81, 1.53)	1.09 (0.78, 1.53)	1.19 (0.88, 1.60)	1.28 (0.93, 1.77)	1.05 (0.76, 1.44)	*1.03 (0.73, 1.44)*
**Smoking at home**								
Up to 20 cig/day	1.04 (0.83, 1.29)	1.11 (0.88, 1.41)	1.27 (0.90, 1.78)	1.26 (0.87, 1.81)	0.88 (0.62, 1.23)	1.04 (0.72, 1.49)	1.28 (0.90, 1.81)	*1.38 (0.96, 2.00)*
≥ 20 cig/day	1.32 (1.00, 1.74)	1.19 (0.87, 1.62)	1.71 (1.11, 2.57)	1.47 (0.90, 2.32)	1.04 (0.66, 1.58)	0.95 (0.57, 1.53)	1.74 (1.13, 2.63)	*1.75 (1.09, 2.76)*
**Maternal smoking during pregnancy**	1.74 (1.26, 2.38)	1.55 (1.08, 2.17)	1.60 (0.96, 2.52)	1.10 (0.63, 1.84)	1.74 (1.02, 2.80)	1.84 (1.04, 3.11)	1.08 (0.60, 1.81)	*0.78 (0.42, 1.36)*
**Family history**	2.82 (2.30, 3.46)	2.78 (2.23, 3.45)	3.12 (2.34, 4.41)	3.50 (2.51, 4.88)	3.64 (2.68, 4.96)	3.75 (2.71, 5.22)	3.34 (2.43, 4.60)	*3.65 (2.60, 5.11)*
	***Children 13–14 years of age***							
	**Current wheeze**		**Severe asthma**		**Current eczema**		***Current Rhinoconjuctivitis***	
	**Unadjusted OR (95%CI)**	**Adjusted OR**‡ **(95% CI)**	**Unadjusted OR (95% CI)**	**Adjusted OR**‡ **(95% CI)**	**Unadjusted OR (95% CI)**	**Adjusted OR**‡ **(95% CI)**	**Unadjusted OR (95% CI)**	***Adjusted OR****‡****(95% CI)***
**Parental education**								
Both secondary	0.98 (0.72, 1.34)	1.02 (0.73, 1.42)	1.03 (0.67, 1.58)	1.10 (0.69, 1.76)	0.74 (0.48, 1.15)	0.83 (0.51, 1.34)	0.63 (0.48, 0.84)	*0.76 (0.56, 1.04)*
At most one tertiary	1.25 (0.85, 1.82)	1.12 (0.73, 1.69)	1.30 (0.75, 2.19)	0.99 (0.52, 1.81)	0.59 (0.31, 1.07)	0.58 (0.29, 1.12)	0.65 (0.44, 0.94)	*0.71 (0.46, 1.08)*
Both tertiary	1.11 (0.72, 1.68)	1.09 (0.68, 1.72)	1.26 (0.69, 2.22)	1.24 (0.63, 2.33)	0.56 (0.28, 1.07)	0.60 (0.27, 1.24)	0.69 (0.45, 1.02)	*0.90 (0.57, 1.40)*
**Animals at home**	1.11 (0.87, 1.41)	1.10 (0.85, 1.44)	1.14 (0.81, 1.61)	1.11 (0.76, 1.63)	2.00 (1.36, 2.99)	2.35 (1.54, 3.65)	1.28 (1.02, 1.62)	*1.40 (1.08, 1.82)*
**Smoking at home**								
Up to 20 cig/day	1.33 (1.01, 1.75)	1.28 (0.96, 1.71)	1.38 (0.94, 2.03)	1.36 (0.90, 2.06)	1.26 (0.83, 1.93)	1.12 (0.72, 1.75)	1.44 (1.10, 1.89)	*1.37 (1.03, 1.83)*
≥ 20 cig/day	1.87 (1.28, 2.71)	1.57 (1.04, 2.32)	1.85 (1.06, 3.10)	1.58 (0.87, 2.77)	2.35 (1.38, 3.92)	1.81 (1.02, 3.13)	2.96 (2.11, 4.13)	*2.73 (1.91, 3.89)*
**Active smoking**	4.88 (2.64, 8.48)	4.49 (2.17, 8.64)	5.77 (2.63, 11.28)	6.03 (2.46, 13.17)	3.92 (1.50, 8.51)	2.71 (0.78, 7.07)	3.20 (1.63, 5.79)	*2.60 (1.15, 5.27)*
**Family history**	3.17 (2.40, 4.15)	3.25 (2.42, 4.33)	4.39 (3.04, 6.30)	5.07 (3.43, 7.45)	2.19 (1.40, 3.33)	2.17 (1.36, 3.37)	2.41 (1.82, 3.16)	*2.26 (1.67, 3.03)*

† Adjusted for each other as well as community (G/C and T/C), gender, parents’ nationality, area of residence, number of siblings, mode of delivery, birth weight, exclusive breastfeeding duration and bedroom sharing.

‡ Adjusted for each other as well as community (G/C and T/C), gender, parents’ nationality, area of residence and number of siblings. Maternal smoking during pregnancy, mode of delivery, birth weight and exclusive breastfeeding duration were not available for the 13–14 year-old children.

### Association of family history of allergy with asthma and allergic symptoms

The markedly lower frequency of family history reported in the T/C community is perhaps suggestive that there might have been underestimation, and thus misclassification in the case of T/C, in which case we would expect that the strength of association between the study outcomes and family history would be weaker in T/C. At least in the younger age-group, adjusted associations of all outcomes with family history of allergies were consistently stronger among the G/C community. Nevertheless, we found no statistical evidence of effect modification by community on the association of family history of allergy with asthma and allergic symptoms (Table 6).

**Table 6:** Adjusted odds ratios of asthma and allergic symptoms in relation to family history of allergies in children from the Greek-Cypriot and Turkish-Cypriot community

	**7-8 year olds**			***13-14 year olds***		
	**G/C**	**T/C**		**G/C**	**T/C**	
	**Odds ratio†**	**Odds ratio**	**p-value**	**Odds ratio‡**	**Odds ratio**	**p-value**
	**(95% CI)**	**(95% CI)**	**LRT§**	**(95% CI)**	**(95% CI)**	**LRT§**
**Outcomes**						
Current wheeze	3.40	2.40	0.12	3.95	2.84	0.27
	(2.42, 4.77)	(1.80, 3.19)		(2.51, 6.21)	(1.92, 4.13)	
Severe asthma	5.47	2.91	0.09	5.90	4.75	0.61
	(2.87, 10.43)	(1.93, 4.33)		(2.92, 11.93)	(2.95, 7.51)	
Current eczema	3.96	3.56	0.74	1.54	2.82	0.19
	(2.48, 6.31)	(2.24, 5.60)		(0.76, 3.12)	(1.53, 4.94)	
Current rhinoconjuctivitis	5.12	3.17	0.21	1.87	2.52	0.34
	(2.68, 9.79)	(2.11, 4.73)		(1.13, 3.07)	(1.73, 3.62)	

† Adjusted for gender, parents’ nationality, parents’ level of education, area of residence, number of siblings, passive smoking, animals at home, maternal smoking during pregnancy, mode of delivery, birth weight, exclusive breastfeeding duration and bedroom sharing.

‡ Adjusted for gender, parental nationality, parental level of education, area of residence, number of siblings, passive smoking, and animals at home.

§ Likelihood ratio test comparing models with and without interaction term between community (G/C & T/C) and family history of allergies.

## Discussion

This is the first study to present estimates of the prevalence of allergic disorders in the two communities of Cyprus that have been living apart on either side of the division line on the island for nearly 40 years. In the G/C community, prevalence of current wheeze in 7–8 year-old children assessed with the use of ISAAC questionnaire was 8.7% in 2008, demonstrating a rising trend from the previous recorded estimate of 6.9% in 2000–01 [12]. In the T/C community, there is currently no evidence regarding the temporal prevalence trends of allergic conditions in childhood. The literature is restricted to a single study, performed in 1999, when prevalence of current wheeze among children aged 6–14 years was estimated at 4.8% [13]. Although the ISAAC protocol was also employed in this study, no direct comparisons can be made with our findings due to the different age spectrum of the study populations.

The main strength of our report is the use of the same standardized protocol to study concurrently childhood allergies in large population samples from two communities that live apart on a small divided island. However, due to the cross-sectional nature of the study and absence of objective markers of atopy no causative inferences can be made between the identified risk factors and study outcomes. The response rate among the G/C community was lower than the one in the T/C community, possibly as a result of the steady rise in the number of surveys performed in G/C schools in the last decade. Nevertheless, we do not think that this has compromised the representativeness of the G/C sample since the socio-demographic characteristics of the participants compare very favorably with the profile recorded in other epidemiological studies performed at a similar time in this population with much higher response rates; details of this, and other sensitivity analyses performed to assess the possible magnitude of selection bias in the G/C sample were published in a previous report [12]. Even though the socio-demographic composition of the G/C sample supports its representativeness, we cannot entirely rule out that the findings are not affected by the lower participation among G/C children. Differential selection bias (whereby allergic G/C children were less likely to participate in the study than T/C allergic children) would result in an underestimation of the true prevalence in the G/C community. However, it is more likely that affected children from both communities were probably more likely to have an incentive to participate in the survey. In that case, the lower response among the G/C would have led to an overestimation of the prevalence rates, and hence, differences in the prevalence of symptoms between the two communities may be even larger than those observed.

The fact that the T/C community presumably leads a less “westernized” lifestyle than the G/C community, made us speculate that the prevalence of allergies may be lower amongst T/C children. Refuting our original hypothesis, our results showed that the prevalence of allergic disorders, with the exception of eczema, was consistently lower in the G/C community, which can not accounted for by the socio-demographic and lifestyle characteristics of the participants.

In contrast, lower prevalence of self-reported family history of allergies was found in the T/C community, suggesting a paradox of a community with lower prevalence of family history of allergy among parents but increased risk of allergies among children. Although such a possibility can not be excluded, it is generally accepted that parental allergy is associated with increased risk of allergies in the offspring [17,18]. It is likely that allergic diseases were underdiagnosed among family members of the T/C participants. Perhaps, this may be the consequence of lower access of the previous generation of T/C to specialized primary care and, as a result, under-detection of allergic diseases or, alternatively, it may be the result of using other diagnostic terms for asthma, eczema or rhinoconjuctivitis in the T/C community at the time. Such a possibility can not be ruled out as in a recent national multicenter study for allergic diseases in schoolchildren in Turkey [19] there was a substantial discrepancy in prevalence between lifetime wheezing ( range 31–37,9% ) and lifetime asthma diagnosed by physician (1.8-6.3%). Despite the possible misclassification in the T/C community, there was no statistical evidence of a weaker association between self-reported family history of allergy and symptoms in the T/C community. We believe that the possible discrepancies in diagnostic labeling between the two communities in Cyprus do not affect the estimates of our study on the prevalence of allergic disorders as we focused on answers to questions of the ISAAC questionnaire referring on symptoms and not on diagnostic labeling.

In contrast to the pattern observed in the case of asthma and hay fever, eczema was more frequent in the G/C community. Although in most European countries high prevalence of eczema is observed along with high prevalence of wheezing [20,21], this is not always the case. In Sweden for instance, the prevalence of reported eczema [20] is considered among the highest in the world (35,7%) whereas the prevalence of asthma [21] is rather moderate (8%).

Our prevalence estimates are generally within the range observed in other countries of the region such as Greece [15] and Turkey [19] The prevalence of current wheeze in Greece ranged from 5.7% to 8.7% in 2001 [15] while in Turkey in 2005 estimates were as high as 14.1% to 22.6% [19], despite the fact that westernized lifestyle is more prominent in Greece. At least with respects to wheeze, estimates from Turkey appear even higher than the prevalence observed among T/C children in our study. The interaction of genetic predisposition with environmental exposures has been shown to influence immune responses relating to asthma pathogenesis and might be of particular importance especially in early life [22,23]. Perhaps genetic susceptibility or unknown environmental risk factors underlie the observed difference between the Greek and Turkish populations and might also be responsible for the observed higher prevalence of atopic symptoms in the T/C children in our study.

Differences between the younger and older age groups in terms of identified risk factors were mainly observed with regards to (a) the association with number of siblings (i.e. restricted to the younger children), (b) area of residence (with lower prevalence of wheeze in rural areas among older children only) and (c) exposure to tobacco smoke (with higher risk of all study outcomes in older children but only rhinoconjunctivitis).

A higher number of siblings seems to protect the younger children against wheezing and eczema but no similar effect was observed in the older group. In contrast, family size was not associated with the risk for rhinoconjuctivitis in the younger group, but increased the risk by 40% in the older group. Most epidemiological studies reported an inverse association between number of siblings and eczema, asthma and more consistently with hay fever [24,25]. However, Matheson et al. [26] showed that number of siblings were important predictors only for the development of early onset allergic rhinitis (<7 years of age) but not for later onset allergic rhinitis perhaps explaining the lack of inverse associations in the older children.

Other hygiene hypothesis factors such as early nursery attendance and contact with farm animals at home were not associated with any allergic disorder although the latter could be attributed to the small number of families (260/10156) who kept farm animals at home. Thus, the protective effect for asthma conferred in older children residing in rural areas cannot be attributed to exposure to farm animals but it is more likely reflecting adverse environmental exposures in children residing in urban areas instead, such as high traffic-related emissions, or the generally more westernized lifestyle of the urban population. It is interesting that no similar urban–rural differences were observed in the younger age-group. A recent study by Kolokotroni et al. [12] showed that, at least in G/C community, there was an increase in the prevalence of wheeze and hay fever between 2000 and 2008 that occurred primarily in the rural rather than the urban areas. It is possible that environmental and lifestyle changes experienced by both communities in the past decade might have led to increasing “urbanization” of rural areas affecting primarily the younger cohort of children. Similarly, in the case of older children the prevailing environmental and lifestyle factors in the rural areas during the first years of their life might have had a protective effect that is extended until adolescence.

In line with the evidence provided by Bruke et al. [27] in a recent meta-analysis we found strong association of wheeze and maternal smoking during pregnancy in the younger children for whom information was available. Exposure to tobacco smoke at home was also associated with at almost 60% increase in the risk of wheezing, nearly doubling the risk for eczema and three times for rhinoconjuctivitis, but only among older children. In younger children, the effect was apparent only for rhinoconjunctivitis. A meta-regression study found that older children exposed to second hand tobacco smoke were more likely than younger children to develop asthma, hypothesizing that the risk increases in later childhood as a result of a longer exposure to smoke [28]. Interestingly, after we stratified the analysis by community, we also observed a significant risk of wheezing with passive exposure to tobacco smoke amongst the younger T/C, but not in the G/C children. This finding may reflect a changing pattern in smoking habits at home amongst the younger generation of parents in the G/C but not in the T/C community (i.e. outdoors instead of indoors).

Active smoking among the 13–14 year olds was not common as it was reported in less than 2% in both communities. Nevertheless, active smoking in our study was a significant risk factor for wheeze and severity of asthma as well as for rhinoconjuctivitis, even after adjusting for passive household tobacco exposure, consistent with the findings of previous studies [29-31].

Finally, we did not observe any clear socio-economic gradient in allergic symptoms, at least when examining parental education as a surrogate measure of socioeconomic status. Current evidence on the impact of socio-economic status on development of allergies is rather conflicting [32-36] with some studies indicating a social gradient for allergic disorders in childhood [33,35] and others not [36].

## Conclusions

Allergic disorders are encountered rather commonly in both communities of Cyprus while it seems that, in contrast to our original speculation driven by the hygiene hypothesis, it appears that the higher burden for asthma and rhinoconjuctivitis in the T/C children cannot be explained by a less westernized lifestyle. The extent to which genetic susceptibility or other environmental factors, not accounted for in this study, account for the observed differences is not known.

## Abbreviations

aOR: Adjusted odd ratio(s); CI: Confidence intervals; G/C: Greek Cypriot; ISAAC: International study of asthma and allergy in childhood; LRT: Likelihood ratio tests; OR: Odd ratio(s); T/C: Turkish Cypriot.

## Competing interests

All authors declare that they have no competing interest(s) that could interfere with their judgment in analyzing and interpreting the findings of this study.

## Authors’ contributions

PY conceived and secured funding for the current study. PY, DM and KA have made contributions to the design of the study. PY supervised and OK coordinated the data collection phase of the study in the G/C community and HK supervised and MF coordinated the data collection phase of the study in the T/C community. DL and MF compiled the data. DL performed the statistical analyses. DL and MM wrote the first draft of this paper. NM supervised the statistical analyses, helped drafting and critically revised the manuscript. All authors assisted in the interpretation of results, revised critically and contributed intellectually towards the final version. All authors read and approved the final manuscript.

## Pre-publication history

The pre-publication history for this paper can be accessed here:

http://www.biomedcentral.com/1471-2458/13/585/prepub
